# Circulating Tumor Cells (CTCs): A Unique Model of Cancer Metastases and Non-invasive Biomarkers of Therapeutic Response

**DOI:** 10.3389/fgene.2021.734595

**Published:** 2021-08-25

**Authors:** Jialing Liu, Jingru Lian, Yafei Chen, Xin Zhao, ChangZheng Du, Yang Xu, Hailiang Hu, Hai Rao, Xin Hong

**Affiliations:** ^1^Department of Biochemistry, School of Medicine, Southern University of Science and Technology, Shenzhen, China; ^2^Department of Hepatobiliary Surgery, The Second Affiliated Hospital and School of Medicine, Southern University of Science and Technology, Shenzhen, China

**Keywords:** circulating tumor cells, cancer metastasis, non-invasive biomarker, therapeutic response, liquid biopsy, microfluidic engineering

## Abstract

Late-stage cancer metastasis remains incurable in the clinic and is the major cause death in patients. Circulating tumor cells (CTCs) are thought to be metastatic precursors shed from the primary tumor or metastatic deposits and circulate in the blood. The molecular network regulating CTC survival, extravasation, and colonization in distant metastatic sites is poorly defined, largely due to challenges in isolating rare CTCs. Recent advances in CTC isolation and *ex vivo* culture techniques facilitates single-cell omics and the development of related animal models to study CTC-mediated metastatic progression. With these powerful tools, CTCs can potentially be used as non-invasive biomarkers predicting therapeutic response. These studies may open a new avenue for CTC-specific drug discoveries. In this short review, we aim to summarize recent progress in the characterization of CTCs and their clinical relevance in various cancers, setting the stage for realizing personalized therapies against metastases.

## Introduction

Cancer remains the leading cause of death worldwide, with the number rolling up to approximately 10 million in 2021, which is expected to be further doubled by the year 2040 (Sung et al., 2021). These striking numbers suggest a pressing need to obtain a comprehensive understanding of cancer etiology and the underlying molecular mechanisms that drive metastasis. The detection and treatment of malignant cancers remain largely ineffective due to inter-patient and intra-tumor heterogeneity, as well as the abilities of cancer cells to disseminate locally or distantly through metastatic progression, resulting in treatment failure and high mortality (Nguyen et al., 2009; Hanahan and Weinberg, 2011). Therefore, there is dire need for disruptive medical technologies that can achieve more reliable cancer diagnosis, higher therapeutic efficacies, and more favorable long-term clinical benefit.

Cancer metastasis is considered a multi-step process involving remodeling of distinct key signaling pathways at each stage (Nguyen et al., 2009; Lambert et al., 2017). They include (1) local invasion of primary tumor cells into neighboring sites; (2) intravasation: migration into nearby blood vessels named “intravasation”; (3) transient traveling and survival in the blood circulation, a population known as “circulating tumor cells (CTCs)”; (4) extravasation: CTCs travel out of the blood vessel and seeding in distant organs, a process named “extravasation”; (5) colonization and growth in the distant sites, that eventually become clinically detectable metastatic tumors. During this process, many metastatic lesions may be present as micrometastases for a long time, which is difficult to detect clinically, yet they appear to be responsible for the eventual disease relapse (Pantel et al., 2009). The therapeutic development that can selectively and effectively target metastatic disease has been limited since tumors are constantly evolving with dynamic genetic and epigenetic alterations during the transitions from a primary tumor to metastases. Although primary tumor biopsies are relatively accessible, they offer very limited information on the behaviors of metastatic tumors, which are often difficult to obtain by surgery. Unfortunately, most of our current treatment strategies are based on the molecular and pathophysiological information derived from primary tumors that may have limited efficacies on metastatic populations due to significant differences between these two groups. Furthermore, remarkable metastatic heterogeneity exists when tumors shed into circulation followed by colonization to distant organs with distinct microenvironment, which makes it extremely challenging to devise a common treatment strategy to simultaneously eliminate different metastatic populations (McGranahan and Swanton, 2017; Hunter et al., 2018). In this context, CTCs, a unique group of cancer cells generated by tumors shedding from primary or metastatic deposits and enter blood circulation, are potential metastatic precursors that give rise to distinct metastatic populations. CTCs could be non-invasively accessible through simple blood draws and thus may offer as a real-time “liquid biopsy” for probing the biological evolutions of cancer during its transition towards metastases (Haber and Velculescu, 2014; Pantel and Speicher, 2016; Alix-Panabieres, 2020).

CTCs are extremely rare, about one in a billion blood cells may be a CTC. This has imposed a significant technical challenge to the efficiently isolate CTCs. The detection and enrichment technologies of CTCs can be broadly categorized into two types (Yu et al., 2011; Ferreira et al., 2016). The first one is positive selection, mostly relying on antibodies capturing the surface tumor antigen expressed on CTCs. One representative example is the CellSearch System utilizing Epithelial Cell Adhesion Molecule (EpCAM)-based CTC capture and enumeration (Riethdorf et al., 2007). Other positive selection-based CTC enrichment technologies involving antibody or peptide-functionalized nanoparticle-based CTC capture followed by fluorescent imaging and enumeration (Li et al., 2019). Although antigen-based CTC detection approaches are relatively sensitive, they may miss a significant fraction of CTC population with the change in expression of some antigens (like EpCAM) during epithelial-to-mesenchymal transitions in breast CTCs (Yu et al., 2013; Papadaki et al., 2019). Thus, antigen-independent negative selection methods appear to offer better coverage of these CTC populations. The negative selection methods are designed to achieve efficient depletion of blood components such as red blood cells, platelets, and white blood cells, using size-based filtration methods or immuno-magnetic depletion using antibodies against common leukocyte markers (Kallergi et al., 2016; Banko et al., 2019). The emergence of several microfluidic-based isolation platforms that can integrate multiple physical and biochemical properties of CTCs into one device allows efficient isolation of CTCs in several cancer types (Jackson et al., 2017; Lin et al., 2020; Pei et al., 2020). One representative example is the CTC-iChip microfluidic system invented by researchers from the Massachusetts General Hospital (Ozkumur et al., 2013; Karabacak et al., 2014). Due to the highly efficient depletion of antibody-tagged white blood cells and size-based sorting strategy, individual viable CTCs and CTC clusters from multiple cancer types could be recovered by the CTC-iChip system in an antigen-independent manner. Isolated CTCs could be subjected for *ex vivo* culture or *in vivo* passage in immunocompromised mice, forming CTC cell lines and CTC-derived animal explant models (CDX), respectively, which serve as powerful tools for mechanistic investigation of CTC biology (Yu et al., 2014; Drapkin et al., 2018; Hong et al., 2021).

CTCs are thought to be pre-metastatic populations that give rise to distant metastases, thus a complete understanding of CTC biology may hold the promise for devising new therapeutic strategies for treating metastatic disease. On the contrary, primary tumor biopsy analyses may only reveal signatures that are confined to local tumors, which may have undergone dramatic molecular remodeling during metastatic progression. Second, tissue biopsies are generally limited by surgical accessibilities while CTC collection can be achieved through a simple blood draw in patients. Since such analyses may be repeated before and after therapies in a longitudinal manner to monitor disease progression and therapeutic response, CTC-based liquid biopsy technologies could potentially enable a highly precise and personalized cancer treatment. Third, a single tumor biopsy often does not represent the whole tumor heterogeneities, which represent a significant challenge leading to disease relapse. On the other hand, CTCs collected from distinct treatment stages may reveal the evolving tumor characteristics and potentially capture the dynamic evolution of the tumor, which may provide important clues in devising more effective cancer therapies. While other liquid biopsies, such as circulation tumor DNA (ctDNA) and exosomes, may similarly serve the purpose in guiding therapeutic decisions during cancer detection and treatment (Pantel and Alix-Panabieres, 2019; Kilgour et al., 2020; Pessoa et al., 2020; Ignatiadis et al., 2021; Yu et al., 2021), CTCs appear to be unique for their functional role as the major metastases-seeding cells. In the next sections, we aim to provide a snapshot of the recent advances in CTC biological studies and how these new findings may be translated into clinical applications.

## Biological Analyses of Circulating Tumor Cells

Although immunostaining and enumeration of CTCs remain effective in predicting disease progression and therapeutic response in a variety of cancer types (Cristofanilli et al., 2004; Sastre et al., 2008; Scher et al., 2009; Riethdorf et al., 2010; Cristofanilli et al., 2019), it is the recent technological revolutions on microfluidic engineering techniques and single-cell omic studies that begin to unravel the molecular “dark matter” of these “metastatic precursor” populations. Using high-resolution single-cell analyses, it is now possible to systematically profile the genome, transcriptome, and proteome of individually isolated CTCs or established CTC lines, which may provide remarkable insights into CTC-mediated metastatic process (Krebs et al., 2014; Micalizzi et al., 2017).

Genomic and transcriptomic studies using single-cell RNA-seq have revealed considerable heterogeneities among individual CTCs and CTC subpopulations, in a manner that may be reflecting the multi-clonal origin of their matched tumors (Lohr et al., 2014; Miyamoto et al., 2015; Jordan et al., 2016; Lim et al., 2019; Riebensahm et al., 2019; Klotz et al., 2020; Hong et al., 2021). The Love lab performed a proof-of-concept study using whole-exome sequencing to characterize the genomic alterations of CTCs from metastatic prostate cancer patients and revealed both common and distinct mutational signatures by comparing CTCs with matched tumors, which may reflect the evolutionary dynamics during metastatic progression (Lohr et al., 2014). Miyamoto et al. carried out single-cell RNA-seq using 77 isolated prostate CTCs from 13 patients and uncovered striking heterogeneities within distinct CTC subpopulations, which contained acquired distinct androgen receptor (AR) gene mutations and differential splice variants expression. Molecular analyses of these cells further identified the correlation between the activation of non-canonical WNT signaling in CTC subclones and therapeutic resistance in patients treated with AR inhibitors (Miyamoto et al., 2015). Hodgkinson et al. (2014) established a panel of small cell lung cancer (SCLC)-derived explant (CDX) models for individualized drug testings, an approach that was also reported in *ex vivo* cultured breast CTC lines (Yu et al., 2014). Using breast CTC lines as well as fresh CTCs isolated from breast cancer patients that were initially diagnosed to be estrogen receptor-positive (ER +) and human epidermal growth factor 2 negative (HER2-), Jordan et al. (2016) identified heterogeneous CTC populations that co-existed as either HER2^+^ or HER2- subclones. Remarkably, these two CTC subtypes were driven by distinct oncogenic signaling and can be inter-convertible upon differential treatment. Simultaneous targeting of both HER2^+^ and HER2- CTC populations using inhibitors blocking both pathways resulted in the abolishment of CTC-mediated tumorigenesis in mouse xenografts (Jordan et al., 2016). Using patient-derived CTC cell lines and mouse models, Klotz et al. (2020) identified Semaphorin 4D and Myc as site-specific drivers of brain metastases. Similarly, biological characterization of five established melanoma CTC lines and single-cell transcriptomic profiling of 76 primary CTCs isolated from 22 metastatic melanoma patients revealed the upregulation of SREBP-mediated lipogenesis in a subset of CTCs with higher proliferative potential, which was well-correlated with drug resistance and poor prognosis (Hong et al., 2021). These studies collectively demonstrated the power of CTC biological characterizations at single-cell levels in deciphering cancer heterogeneities and metastatic tropisms (Keller and Pantel, 2019).

**FIGURE 1:**
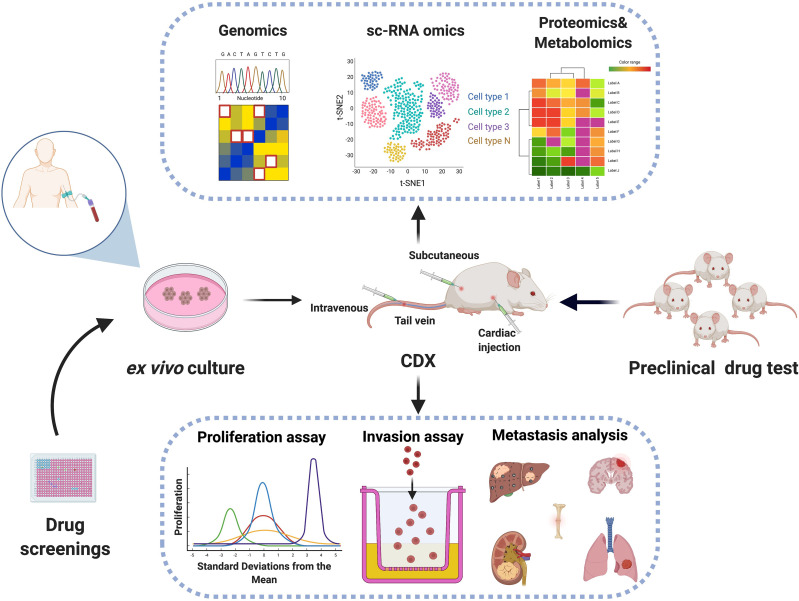
Biological characterization of CTCs using CTC-based model systems. Viable CTCs may be isolated from patient blood samples followed by *ex vivo* expansion in plates and *in vivo* inoculation in immunocompromised mice. The establishment of CTC cell lines and CTC-derived mouse explants (CDX) may be coupled with genomic, transcriptomic, proteomic, or metabolomic technologies for dissecting mechanisms of metastasis and served as preclinical drug test platforms.

It was initially discovered that some breast cancer patients had CTCs traveled along with white blood cells within the circulation called “CTC clusters” (Stott et al., 2010; Yu et al., 2013). Elegant studies coupling single-CTC profiling and mouse models have subsequently provided significant biological insights on these very rare but clinically important CTC subpopulations (Aceto et al., 2014; Gkountela et al., 2019; Szczerba et al., 2019; Donato et al., 2020). DNA methylome analyses of single CTC and CTC clusters uncovered hypomethylated regions that were selectively occupied by the key stemness transcription factors OCT4, NANOG, SOX2, and SIN3A, which may play critical roles in the metastatic seeding of CTC clusters (Gkountela et al., 2019). Further single-cell studies of CTC-associated white blood cells had pinpointed an unexpected role of neutrophils in promoting CTC cluster proliferation and metastatic colonization (Szczerba et al., 2019). Other heterotypic interactions of CTCs with other non-CTC cells such as cancer-associated stromal cells, fibroblasts, and certain immune cell types were also reported to have a significant impact on CTC survival or metastatic colonization (Duda et al., 2010; Labelle et al., 2011; Sprouse et al., 2019; Ortiz-Otero et al., 2020). Therefore, the establishment of CTC experimental systems including *ex vivo*-cultured CTC lines, CDX models, in conjunction with single-cell omic technologies may greatly accelerate the fundamental research on CTC biology, and open the door for the potential implementation of CTC-based clinical applications, such as CTC bio as companion diagnostics and novel therapies that selectively eliminate CTCs (Figure 1).

## Clinical Translations From CTC Biological Discoveries

The enumeration of CTCs has been clinically validated as an important prognosis factor in multiple cancers such as breast, prostate, and colorectal cancers in patients (Cristofanilli et al., 2004; Riethdorf et al., 2007; Sastre et al., 2008; Scher et al., 2009; Miller et al., 2010). However, its clinical utilities remain relatively limited given the vast degree of CTC heterogeneities, variabilities in CTC isolation technologies, and potential biases during CTC downstream molecular processing and manipulation steps that appear to vary from one study to another. Nevertheless, its clinical potential may be fully unleashed at a time when we are able to achieve a complete understanding of the biological properties of CTCs. With the abundance of molecular-level information derived from CTCs at the genomic, transcriptomic, proteomic, and metabolomic levels, CTC-based biomarker panels may be developed to precisely stratify patient and monitor disease monitoring following distinct therapeutic courses.

With the knowledge of cancer or CTC-specific transcriptomic studies, we and collaborators recently developed highly sensitive and specific CTC molecular assays using microfluidic enrichment of CTCs coupled with digital droplet PCR (ddPCR)-based profiling technologies (Kalinich et al., 2017; Hong et al., 2018; Kwan et al., 2018; Miyamoto et al., 2018). Individual transcripts that were abundant in CTCs but barely detectable in healthy donor white blood cells were quantified before and after specific therapies using serial blood collections. Such approaches revealed the predictive value of CTC signatures in several distinct cancer types under different treatment regimens. A 10-gene CTC signature was applied to monitor the therapeutic efficacy of patients bearing hepatocellular carcinoma (HCC) and can be used to detect early HCC occurrence in the high-risk population (Kalinich et al., 2017). In a prospective cohort of metastatic melanoma patients receiving immune checkpoint inhibition therapies, 19-gene CTC RNA signatures were implemented to monitor early treatment response, which showed a strong predictive value of long-term clinical outcome in two independent clinical studies (Hong et al., 2018; Aya-Bonilla et al., 2020). Similarly, a panel of prostate CTC-specific RNA signatures was assessed for its ability to guide treatment selection in both localized and metastatic prostate cancer patients (Miyamoto et al., 2018). Such CTC quantification strategy was also demonstrated to be effective in inferring treatment response in both localized and metastatic breast cancer patients (Kwan et al., 2018).

**FIGURE 2:**
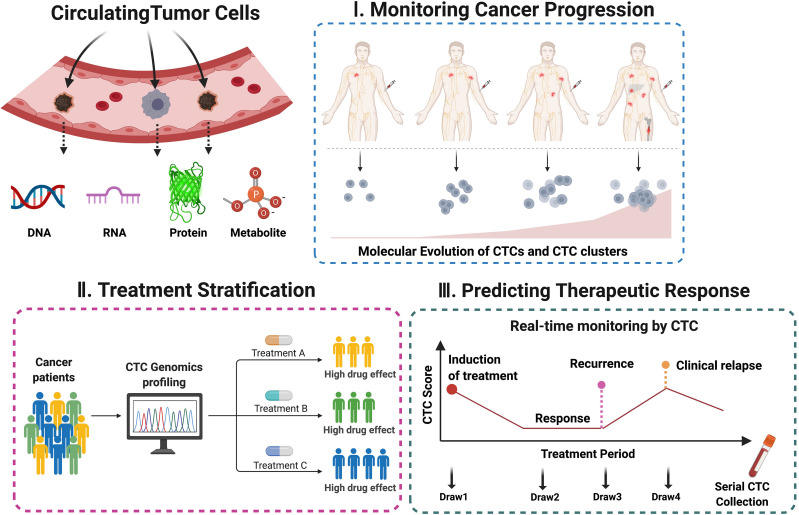
Potential clinical applications using CTC-derived molecular signatures. The molecular and genetic information of CTCs analyzed in a well-defined treatment window may serve as non-invasive biomarker surrogates capable of real-time monitoring of cancer progression, patient stratification, and prediction of long-term clinical outcome.

In addition to RNA-level molecular profiling, quantification of CTC-specific DNA copy number alterations (CNA) was applied to stratify chemo-sensitive and resistant patients in small cell lung cancer (SCLC) (Carter et al., 2017). Direct monitoring of T790M mutation in both CTCs and ctDNAs were employed to potentially guide treatment selection on EGFR-mutant non-small cell lung cancer (NSCLC) patients (Sundaresan et al., 2016). Whole-genome sequencing of single CTCs isolated from NSCLC patients revealed mutational signatures that were shared with metastases that were found 10 months after CTC analyses. Remarkably, these signatures were not detectable in matched primary tumors, suggesting the potential utility of CTC genomic signatures in inferring metastatic propensity and disease relapse (Chemi et al., 2019). Besides these transcriptomic and genomic profiling methods, recent studies have also attempted to perform proteomic or metabolomic characterizations of individual CTCs using microfluidics or mass spectrometry-based technologies (Li et al., 2015; Spitzer and Nolan, 2016; Sinkala et al., 2017; Abouleila et al., 2019; Liu et al., 2019). Although more independent clinical validations with a larger cohort of patient studies are needed, these reports provide the proof-of-principle evidence that CTC molecular signatures may be translated into clinical applications for the accurate stratification of patients, precise monitoring of cancer progression and prediction of therapeutic response throughout the entire treatment courses (Figure 2).

## Conclusion and Future Perspectives

The recent advances in microfluidics enable the efficient isolation of viable CTCs that could be propagated by *ex vivo* culture and *in vivo* animal models (Lallo et al., 2017; Pei et al., 2020). Single-cell sequencing technologies have greatly accelerated the biological discoveries of CTCs. The establishment of CTC-based experimental systems together with single-cell omic profilings will undoubtedly pave the way to decipher the molecular dynamics of CTC-mediated metastatic evolution. Such comprehensive understanding of CTC biology is critically important for devising effective CTC biomarker panels for personalized cancer monitoring and diagnosis, while novel treatment strategies targeting CTCs may represent a new dimension of cancer therapies. Yet, several issues remain to be resolved before CTC liquid biopsies could be truly brought to clinical practice. More efficient and scalable CTC isolation platforms should be developed, so that viable CTCs could be routinely recovered in a variety of solid cancer types, but not just on the few reported cancer types thus far. The success rate of CTC culture and preclinical CDX models remains very low that warrants further improvement before they could be used for longitudinal studies in patients. As a result, the current collections of CTC cell lines or CDX models are still far too limited for conducting systematic drug studies. Further, the sensitivity and specificity of current CTC biomarker panels require substantial improvement. Novel ultra-high throughput quantification strategies that may enable simultaneous profiling of multi-marker panels are yet to be developed to provide comprehensive coverage of these highly heterogenous cancer cell subpopulations. Despite these challenges, the development of CTC-based liquid biopsies is rapidly moving forward, which may dramatically expand our knowledge of cancer metastases and ultimately change the clinical practice of cancer management.

## Author Contributions

JaL and XH conceptualized and wrote the manuscript. All authors contributed to the writing and revision of the manuscript.

## Conflict of Interest

The authors declare that the research was conducted in the absence of any commercial or financial relationships that could be construed as a potential conflict of interest.

## Publisher’s Note

All claims expressed in this article are solely those of the authors and do not necessarily represent those of their affiliated organizations, or those of the publisher, the editors and the reviewers. Any product that may be evaluated in this article, or claim that may be made by its manufacturer, is not guaranteed or endorsed by the publisher.
